# CDC2/SPDY transiently associates with endoplasmic reticulum exit sites during oocyte maturation

**DOI:** 10.1186/1471-213X-9-8

**Published:** 2009-02-03

**Authors:** Jurriaan J Hölzenspies, Willem Stoorvogel, Ben Colenbrander, Bernard AJ Roelen, Dagmar R Gutknecht, Theo van Haeften

**Affiliations:** 1 Department of Farm Animal Health, Faculty of Veterinary Medicine, Utrecht University, Utrecht, the Netherlands; 2 Department of Biochemistry & Cell Biology, Faculty of Veterinary Medicine, Utrecht University, Utrecht, the Netherlands; 3 Department of Reproductive Medicine, University Medical Centre, Utrecht, the Netherlands

## Abstract

**Background:**

Mammalian oocytes acquire competence to be fertilized during meiotic maturation. The protein kinase CDC2 plays a pivotal role in several key maturation events, in part through controlled changes in CDC2 localization. Although CDC2 is involved in initiation of maturation, a detailed analysis of CDC2 localization at the onset of maturation is lacking. In this study, the subcellular distribution of CDC2 and its regulatory proteins cyclin B and SPDY in combination with several organelle markers at the onset of pig oocyte maturation has been investigated.

**Results:**

Our results demonstrate that CDC2 transiently associates with a single domain, identified as a cluster of endoplasmic reticulum (ER) exit sites (ERES) by the presence of SEC23, in the cortex of maturing porcine oocytes prior to germinal vesicle break down. Inhibition of meiosis resumption by forskolin treatment prevented translocation of CDC2 to this ERES cluster. Phosphorylated GM130 (P-GM130), which is a marker for fragmented Golgi, localized to ERES in almost all immature oocytes and was not affected by forskolin treatment. After removal of forskolin from the culture media, the transient translocation of CDC2 to ERES was accompanied by a transient dispersion of P-GM130 into the ER suggesting a role for CDC2 in redistributing Golgi components that have collapsed into ERES further into the ER during meiosis. Finally, we show that SPDY, rather than cyclin B, colocalizes with CDC2 at ERES, suggesting a role for the CDC2/SPDY complex in regulating the secretory pathway during oocyte maturation.

**Conclusion:**

Our data demonstrate the presence of a novel structure in the cortex of porcine oocytes that comprises ERES and transiently accumulates CDC2 prior to germinal vesicle breakdown. In addition, we show that SPDY, but not cyclin B, localizes to this ERES cluster together with CDC2.

## Background

Fully grown immature mammalian oocytes are arrested at the diplotene stage of meiotic prophase I. Oocyte maturation is initiated in vivo when the mural granulosa cells respond to the preovulatory luteinizing hormone surge, or in vitro when oocytes are isolated from follicles [1]. Germinal vesicle breakdown (GVBD) marks the onset of nuclear maturation, which progresses into formation of the first metaphase spindle, followed by extrusion of the first polar body and formation of the second metaphase spindle. At metaphase II, oocytes enter a second period of meiotic arrest, which is maintained until fertilization. Meiosis resumption is often characterized by the occurrence of GVBD, since this is the first clear morphological event that takes place after release from meiotic inhibition. However, extensive rearrangements of components within the ooplasm, known as cytoplasmic maturation [2], already start to occur prior to GVBD [3].

Cytoplasmic maturation includes dynamic changes in the distribution and integrity of the Golgi apparatus and endoplasmic reticulum (ER) [4-6]. In somatic cells, the Golgi apparatus is fragmented at the onset of mitosis and starts to reform at telophase [7]. Two distinct views on the mechanism of Golgi partitioning during mitotis have been proposed [7,8]. One view holds that association of Golgi fragments with the metaphase spindle allows equal partitioning of Golgi components into the two daughter cells [9-11]. The second view is based on the idea of a dynamic Golgi apparatus, in which Golgi proteins continuously cycle through the ER. Coat protein II (COPII)-coated vesicles that traffic from ER to Golgi originate at subdomains of the ER, known as ER exit sites (ERES). Vesicle formation at ERES is inhibited during mitosis as a consequence of which cycling Golgi proteins become trapped in the ER [12]. Golgi components are then equally distributed into daughter cells together with the ER and the Golgi is reformed from vesicles that form at ERES when the ER export block is lifted at telophase [13,14]. Although the general distribution of ER during oocyte maturation has been studied extensively [15], a function for ERES during oocyte maturation remains to be elucidated. Evidence for a role of either of these two mechanisms in the control of Golgi dynamics during oocyte meiosis is lacking. It is clear that cytoplasmic processes constitute an integral part of both mitosis and meiosis, and we therefore use the term 'meiosis resumption' to indicate the moment when the first rearrangement of components occurs within the oocyte in response to release from the inhibitory influence of the follicular environment.

In most cells, cell division cycle 2 (CDC2, also referred to as cyclin-dependent kinase 1) complexes with cyclin B to form M-phase promoting factor (MPF), a well known central regulator of both mitotic and meiotic events. MPF regulates chromosome condensation, nuclear envelope breakdown, and formation of metaphase spindles in both somatic cells and oocytes, whereas transition from metaphase to anaphase requires inactivation of MPF [16]. In mitotic cells, disassembly of Golgi and ERES are regulated by CDC2 through phosphorylation of GM130 and p47, respectively [17,18]. Despite the similarities in CDC2 functions during mitosis and meiosis, it is unclear whether the activity of CDC2 exerts a similarly stringent control over the integrity of the Golgi apparatus and ERES in oocytes as it does in mitotic cells.

The activity of CDC2 during the meiotic divisions of porcine oocytes is known to be controlled by at least two regulatory proteins, i.e. cyclin B and speedy (SPDY) [19,20]. SPDY proteins have no homology to cyclins, but are potent activators of CDC2 during early phases of oocyte maturation [21]. CDC2 activity can alternatively be controlled through phosphorylation of specific residues. Compared to the CDC2/cyclin B complex, the activity of CDC2 when complexed with SPDY is less sensitive to phosphorylation [19]. Finally, CDC2 activity may be regulated by changing the subcellular distribution of CDC2 and/or its regulators [22]: Sequestration of CDC2 to subcellular domains may prevent CDC2 from phosphorylating specific substrates or limit access of regulatory proteins to CDC2. Conversely, concentration of CDC2 at subcellular 'foci', such as the Golgi or ERES, could confer spatio-temporal specificity to CDC2 function.

To establish the role of the subcellular (re-)distribution of CDC2, and its regulatory proteins cyclin B and SPDY, in the regulation of early oocyte maturation, we examined their localization and the distribution of cell organelles containing potential CDC2 target proteins during pig oocyte maturation. The localization of these organelles was investigated using antibodies raised against the ERES marker SEC23, the Golgi marker GM130, and phosphorylated GM130 (P-GM130), a marker for fragmented Golgi. Our results demonstrate that CDC2 transiently associates with a P-GM130-labeled structure in the cortex of maturing porcine oocytes prior to GVBD. Using the ERES marker SEC23, we show that this structure consists of a cluster of ERES. Furthermore, our data on the distribution of CDC2 and its regulatory protein SPDY in oocytes suggest a role for the CDC2/SPDY complex in regulation of secretion during oocyte maturation.

## Results

### CDC2 accumulates in a single cortical structure in GV stage oocytes

**Figure 1:**
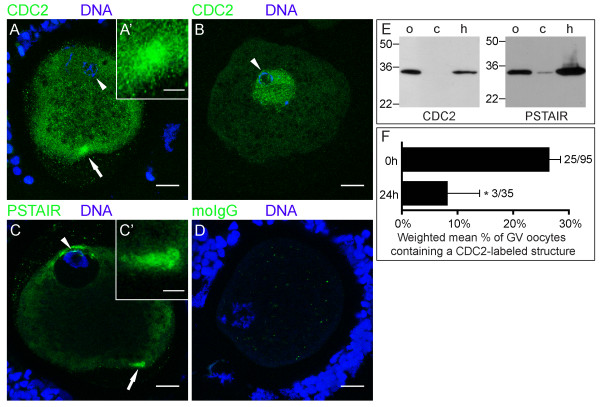
**CDC2 accumulates in a single cortical structure in GV stage oocytes**. (A, B) 0 h GV stage oocytes labeled for CDC2 (green) and DNA (blue) showing CDC2-accumulation in the cortex of the oocyte (A) and in the GV (B). (C) 0 h GV stage oocyte showing PSTAIR labeling (green) and DNA (blue). PSTAIR also accumulates in a cortical domain. Arrows indicate the area that is shown enlarged in the insets (A', C'), and arrowheads denote the GV. (D) 0 h GV stage oocyte from the same experiment as the oocyte in A, labeled with mouse IgG (moIgG; green) and DNA marker (blue). The image was produced using the same confocal settings and enhancements as in A. All images are Z-projections of 3 consecutive confocal sections. Scale bars represent 20 μm in A-D, and 5 μm in A' and C'. (E) Western blot of CDC2 and PSTAIR on ~250 oocytes (o) and cumulus cells (c) collected during the denuding procedure of these oocytes showing a specific band at ~34 kDa. A HeLa cell lysate (h) was used as positive control. Molecular weight (kDa) is indicated on the left of each set of lanes. (F) Weighted mean percentage ± weighted SEM of GV stage oocytes that contained a CDC2-labeled cortical domain at 0 and 24 hours of maturation in 3 independent experiments. Absolute numbers are indicated to the right of the bars (number of positive oocytes/total number of oocytes) and * denotes significant difference (P < 0.05).

To determine the subcellular distribution of CDC2, oocytes were fixed after 0 or 24 h of in vitro maturation (IVM), immuno-labeled and analyzed by confocal laser scanning microscopy. At 0 h, we observed a single large CDC2-containing cytoplasmic structure in the cortex of ~25% of GV oocytes (Fig. 1A). High magnification images showed that this CDC2-positive structure was composed of clustered smaller units (Fig. 1A'). Additionally, ~35% of oocytes displayed CDC2 staining in the GV (Fig. 1B), a pattern that rarely coincided with staining in a cortical structure. Staining with an anti-PSTAIR antibody (Fig. 1C, C'), which recognizes the cyclin-binding domain that is conserved in all CDKs, including CDC2 [23], showed a morphologically identical structure. As could be expected from a broader specificity, PSTAIR-reactive antibodies also labeled other intracellular areas, e.g. cytoplasmic areas near the GV at the plasma membrane of the oocyte (Fig. 1C). Labeling with control IgG from pre-immune serum revealed sporadic non-specific small puncta (Fig. 1D). Specificity of the CDC2 and PSTAIR directed antibodies was further supported by immunoblotting, which revealed a single specific band at the expected 34 kDa molecular weight in oocyte and HeLa cell lysates. Little if any CDC2 and PSTAIR could be detected in the cumulus cells isolated from cumulus-oocyte complexes, which is probably due to low mitotic activity of these cells (Fig. 1E).

The rate of oocyte maturation in our experimental system was consistent with those previously reported in IVM studies using similar maturation conditions [24]: All oocytes were in GV stage directly after isolation (0 h; n = 103); after 24 h of culture (n = 67), 39 ± 5% of oocytes were still in GV, while 61 ± 5% had progressed to MI or MII; after 44 h of maturation (n = 56), 88 ± 4% of oocytes were in MII stage. The association of CDC2 with the cortical structure depended on the state of oocyte maturation, since the percentage of GV oocytes containing a CDC2-labeled structure was significantly reduced from 26 ± 2% at 0 h to 9 ± 6% at 24 h of IVM (Fig. 1F), while this CDC2-labeled structure was not observed in any oocytes that had progressed to MI or MII. Taken together, these data demonstrate that CDC2 associates with a single cortical structure prior to GVBD and suggest that this association is transient.

### CDC2 associates with ER exit sites

**Figure 2:**
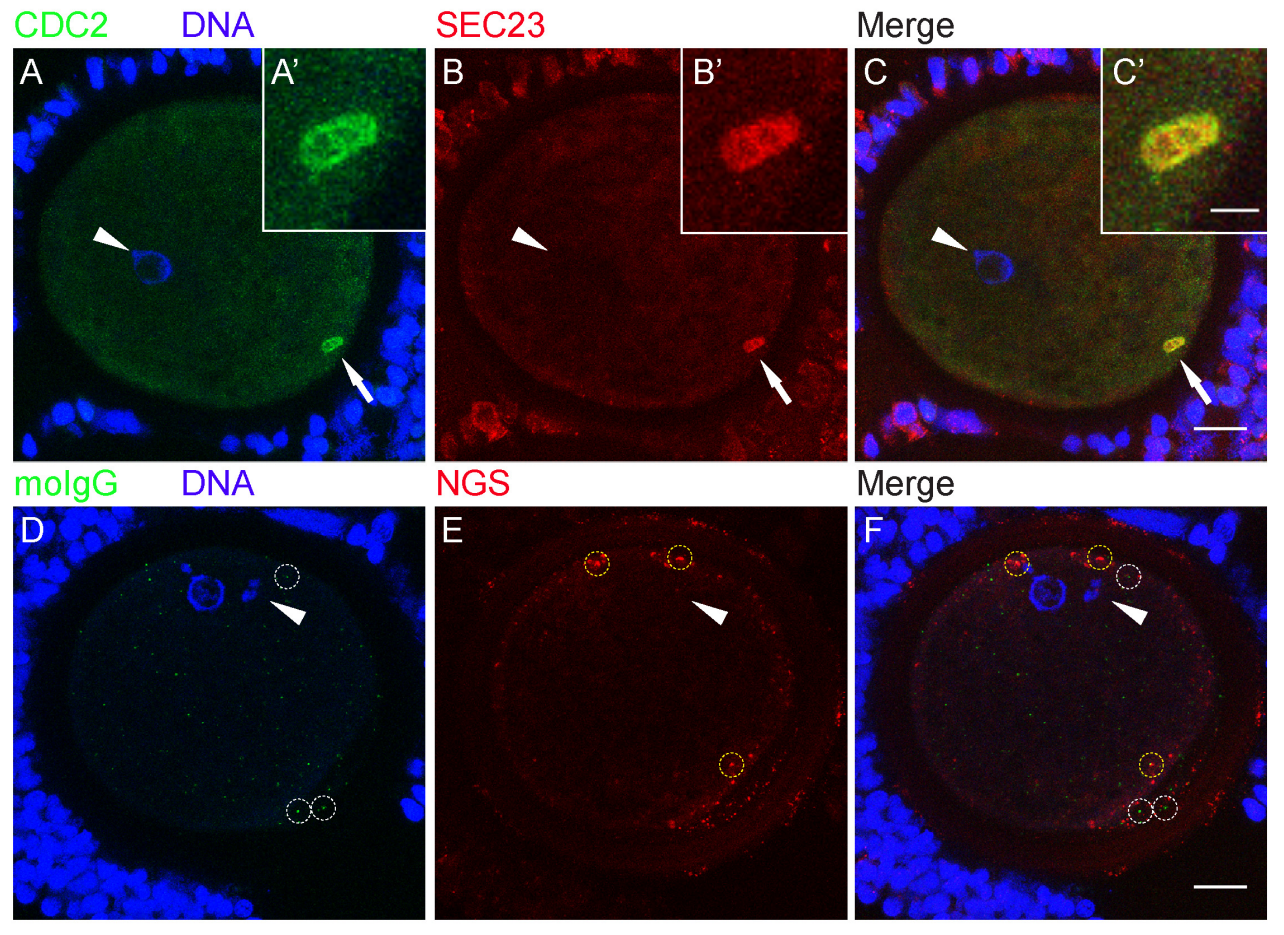
**CDC2 associates with ER exit sites**. (A-C) 0 h GV stage oocyte labeled for CDC2 (A, green), DNA (A, blue), and SEC23 (B, red). Colocalization (yellow) of CDC2 and SEC23 in a cortical domain in the ooplasm is evident in the merged image (C). (D-F) 0 h GV stage oocyte from the same experiment as A-C, labeled with mouse IgG (moIgG, D, green), DNA-marker (D, blue), and normal goat serum (NGS, E, red). Aspecific dots of moIgG staining (examples are marked with white circles in D, F) and NGS (examples are marked with yellow circles in E, F) do not colocalize in the merged image (F). Confocal sections from an acquisition depth equivalent to A-C are shown, and enhancements of D-F were identical to A-C. Images are Z-projections of 6 consecutive sections; scale bars represent 20 μm in C and F, and 5 μm in C'. Arrows indicate the region of the oocyte that is shown enlarged in the insets (A'-C'). Arrowheads denote the position of the GV.

**Figure 3:**
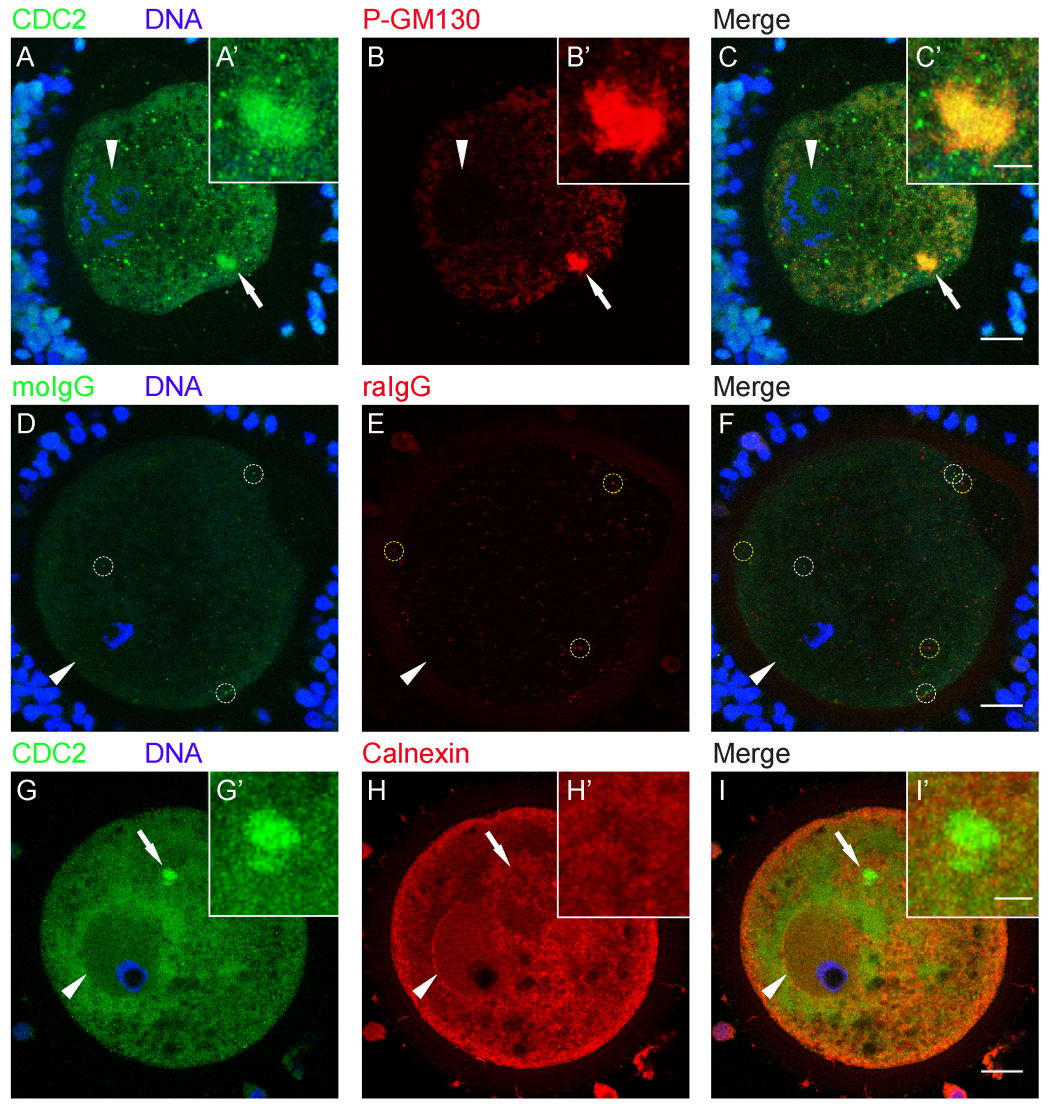
**The fragmented Golgi marker P-GM130 colocalizes with CDC2 at ERES in immature oocytes**. (A-C) 0 h GV stage oocyte labeled for CDC2 (A, green), DNA (A, blue), and phosphorylated GM130 (B, red). Colocalization (yellow) of CDC2 and phosphorylated GM130 in a cortical compartment in the ooplasm is evident in the merged image (C). (D-F) 0 h GV stage oocyte from the same experiment as A-C, labeled with moIgG (D, green), DNA-marker (D, blue), and rabbit IgG (raIgG, E, red). None of the aspecific dots (examples are marked with white circles in D, F and yellow circles in E, F) colocalize in the merged image (F). This control image was subjected to the same enhancements as A-C, and selected to show confocal sections from an equivalent acquisition depth. (G-I) 0 h GV stage oocyte labeled for CDC2 (G, green), DNA (G, blue), and the ER-marker calnexin (H, red). No colocalization (yellow) of CDC2 and calnexin was observed in the merged image (I). Images are Z-projections of 6 consecutive sections (A-F), or 3 consecutive sections (G-I); scale bars represent 20 μm in C, F, and I, and 5 μm in C' and I'. Arrows indicate the region of the oocyte that is shown enlarged in the insets (A'-C' and G'-I'). Arrowheads denote the position of the GV.

**Figure 4:**
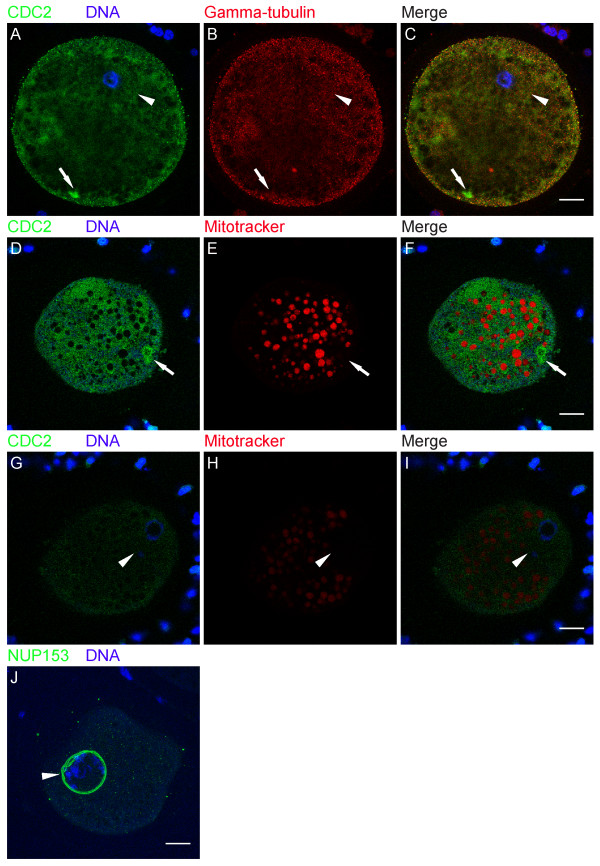
**Gamma-tubulin, mitochondria, and NUP153 do not associate with ERES**. (A-C) 0 h GV stage oocyte labeled for CDC2 (A, green), DNA (A, blue), and gamma-tubulin (B, red). The CDC2-labeled ERES cluster did not label with gamma-tubulin (C). (D-F) 0 h GV stage oocyte labeled for CDC2 (D, green), DNA (D, blue), and mitochondria, using the mitochondrial marker mitotracker (E, red). Mitotracker did not label CDC2-positive ERES (F). (G-I) Confocal section through the same oocyte as in D-F showing the GV. Note that the much lower staining intensity in G-I compared to D-F is solely the result of a ~65 μm change in depth of imaging, since acquisition settings and image enhancement were identical between these images. (J) 0 h GV stage oocyte labeled for NUP153 (green) and DNA (blue). None of the oocytes examined (0/15) showed a cortical domain. The 3 separate double lines around the GV are the result of small size and shape changes between the 3 consecutive sections used to produce the image. Images are either Z-projections of 3 consecutive sections (A-C, J), or single sections (D-I); scale bars represent 20 μm in C, F, I, and J. Arrows indicate CDC2-labeled ERES, and arrowheads denote the position of the GV.

To identify the nature of these CDC2-labeled structures, immature (0 h) oocytes were double-labeled for CDC2 and several other markers: SEC23, GM130, P-GM130, calnexin, gamma-tubulin, NUP153, and mitotracker (see Table 1). SEC23 is a component of the COPII complex, which is involved in the formation of transport vesicles at ERES. COPII-coated vesicles transfer cargo from ER to Golgi and dissociate their coat after fission from ERES. In 0 h GV oocytes, half of the CDC2-labeled structures were positive for SEC23 (Fig. 2A–C; IgG controls are shown in D-F), identifying them as ERES. GM130 is a Golgi matrix protein. At the onset of mitosis, GM130 is phosphorylated by CDC2 resulting in fragmentation of the Golgi apparatus and redistribution of the GM130 protein [27]. Almost all (95 ± 3%) CDC2-positive structures in 0 h GV oocytes also labeled for P-GM130 (Fig. 3A–C; IgG controls are shown in D-F). Calnexin is an ER-resident transmembrane protein, that localizes to the rough ER, but not to ERES [26,30]. Accordingly, we observed calnexin in a reticular pattern and at the nuclear envelope in 0 h GV oocytes (Fig. 3H, I). Consistent with its absence at ERES, calnexin was not found at CDC2-labeled cortical structures in any of the GV oocytes examined (Fig. 3G, I). Finally, gamma-tubulin (Fig. 4A–C), which localizes to microtubule organizing centers, mitotracker (Fig. 4D–I), a marker for mitochondria, and NUP153 (Fig. 4J), one of the nuclear pore complex proteins that localizes to the nuclear membrane and to annulate lamellae, were not observed at CDC2-labeled structures. These results indicate that CDC2 accumulates in a specialized compartment in the smooth ER that comprises ERES.

**Table 1:** Listing of markers used to establish the identity of cortical structures in immature oocytes.

**Name**	**Type**	**Target**	**Reference**	**Present in a cortical structure**
CDC2	mouse monoclonal antibody	Cell division cycle 2, catalytic subunit of MPF	[22]	+
PSTAIR	mouse monoclonal antibody	Cyclin binding domain in all Cdks	[23]	+
SEC23	goat polyclonal antibody	SEC23, component of the coat protein II complex	[18]	+
P-GM130	rabbit polyclonal antibody	GM130 phosphorylated on serine 25	[27]	+
Calnexin	rabbit polyclonal antibody	Calnexin, transmembrane protein	[26]	-
Gamma-tubulin	rabbit polyclonal antibody	Gamma-tubulin, cytoskeletal component	[25]	-
Mitotracker*	Fixable live cell dye	Oxidation within mitochondria	[28]	-
NUP153	mouse monoclonal antibody	Nucleoporin 153 kDa	[29]	-
GM130	mouse monoclonal antibody	Structural Golgi protein	[27]	-
Cyclin B	mouse monoclonal antibody	Cyclin B, regulatory subunit of MPF	[22]	-
SPDY	rabbit polyclonal antibody	SPDY, regulatory protein of CDC2	[19]	+

* Stained by 30 min incubation in maturation medium prior to fixation.

### Inhibition of meiosis resumption prevents association of CDC2 with P-GM130-labeled ERES

**Figure 5:**
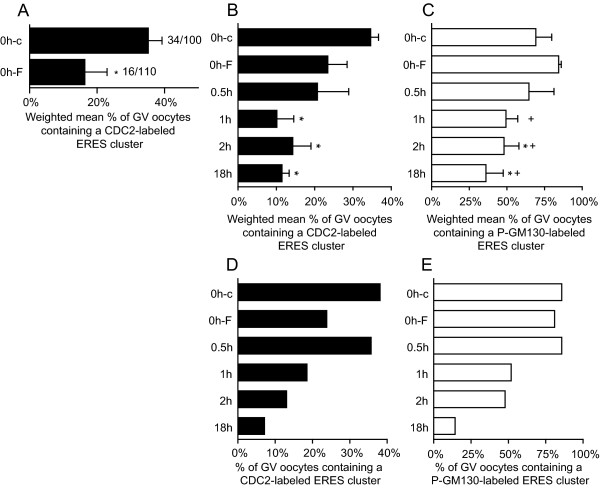
**Inhibition of meiosis resumption prevents association of CDC2 with P-GM130-labeled ERES**. (A) Weighted mean percentage ± weighted SEM of GV stage oocytes containing CDC2-labeled ERES clusters after treatment with either DMSO (0h-c) or 100 μM forskolin (0h-F) during the isolation procedure. Data from 3 separate experiments is shown and * indicates significant difference (P < 0.05). Absolute numbers are shown next to the bars (number of positive oocytes/total number of oocytes). (B, C) Forskolin chase bar graphs (n = 3; 20–30 oocytes per group), showing the weighted mean percentage ± weighted SEM of GV stage oocytes containing a cluster of ERES, labeled for CDC2 (B) and/or P-GM130 (C). In one of the experiments shown, an equal percentage of CDC2-labeled ERES was observed in 0h-F and 0h-c groups. As a result, the difference between 0h-c and 0h-F is significant in A, but not in B. The original dichotomous data (presence/absence of an ERES cluster) was analyzed in SPSS using binary logistic regression. All groups were compared to 0h-c and 0h-F conditions for a total of 10 comparisons per label. The familywise significance level was set to 0.05, and was adjusted for pairwise comparisons using the Holm-Bonferroni procedure. Significant differences compared to 0h-c or 0h-F are indicated by * or +, respectively. (D, E) Representative example of a forskolin chase experiment from the series of experiments shown in B, C, showing the percentage of GV stage oocytes containing a cluster of ERES, labeled for CDC2 (D) and/or P-GM130 (E). Note that the increase in the occurrence of CDC2-labeled ERES at 0.5 h after release from forskolin-maintained inhibition of maturation (D) is obscured in the chart showing combined data from three experiments (B), because it occurred at varying time points. Conditions on the y-axis are: 0 h DMSO control (0h-c), 0 h forskolin (0h-F), and 0.5, 1, 2, and 18 h of maturation after forskolin removal (0.5 h/1 h/2 h/18 h).

In our lab, collecting, selecting, and denuding oocytes requires approximately 2.5 h. Although oocytes in our experimental 0 h condition are still at the GV stage, they may already have resumed meiosis during this experimental interval. To prevent premature meiosis resumption during oocyte isolation, cumulus-oocyte complexes were collected in the presence of 100 μM forskolin [31]. Forskolin stimulates the activity of adenylate cyclase, thus raising the intracellular cAMP concentration. Under these conditions, oocytes are maintained in prophase I arrest [32]. The presence of forskolin during oocyte isolation and denuding significantly reduced the occurrence of CDC2-labeling at the ERES cluster in GV stage oocytes (Fig. 5A), supporting the idea that CDC2 is recruited to ERES early after meiosis resumption. Since maintenance of meiotic arrest by forskolin is reversible [33], oocytes were isolated in the presence of forskolin, and examined at several time points after removal of forskolin from the culture media (forskolin chase). The percentage of GV oocytes containing CDC2-labeled ERES increased within 2 h of chase (Fig. 5B, D), followed by a decline to ~10% after 18 h of chase. In 0 h forskolin treated oocytes, the occurrence of P-GM130-labeled ERES equaled that in 0 h controls. A gradual decline in the percentage of GV oocytes containing P-GM130-labeled ERES was observed after forskolin removal (Fig. 5C, E). Combined, these results indicate that P-GM130 accumulates at ERES prior to meiosis resumption, and that CDC2 transiently associates with ERES just after the oocyte is released from the inhibitory influence of the follicle.

### Phosphorylated GM130 is stored at ERES during maturation

**Figure 6:**
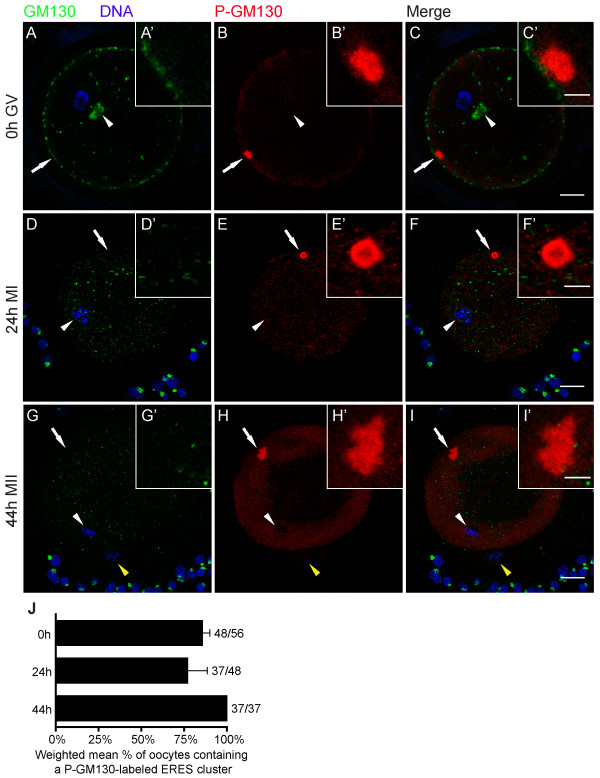
**Phosphorylated GM130 is stored at ERES during maturation**. Oocytes were stained for GM130 (green), P-GM130 (red), and DNA (blue) at different maturation stages. (A-C) 0 h GV stage oocyte containing a perinuclear Golgi apparatus (arrowhead). (D-F) 24 h matured MI oocyte containing a metaphase plate (arrowhead). (G-I) 44 h matured MII oocyte showing the second metaphase plate (arrowhead) and a polar body (yellow arrowhead). Arrows indicate the area that is shown enlarged in the insets. Z-projections of 3 (A-F) or 6 (G-I) consecutive sections are shown; scale bars represent 20 μm in C, F and I, and 5 μm in C' F' and I'. Arrows indicate the region of the oocyte that is shown enlarged in the insets (A'-I'). (J) Weighted mean percentage ± weighted SEM of oocytes that contain a P-GM130-labeled cortical domain at the indicated maturation times. Results from 2 independent experiments are shown.

Given the role of CDC2 in phosphorylating the Golgi protein GM130, labeling for both GM130 and P-GM130 should provide information on the role of CDC2-labeled cortical domains in Golgi-protein redistribution during meiosis. To establish the distribution of GM130 and its phosphorylated form P-GM130 during oocyte maturation, oocytes were double-labeled for GM130 and P-GM130 after 0, 24, and 44 h of IVM. GM130 staining revealed an intact Golgi apparatus and several GM130-labeled areas in the periphery of 0 h GV oocytes, whereas GM130 was not detected in CDC2-labeled ERES (Fig. 6A). As maturation progressed, GM130 staining in the oocyte was observed in progressively smaller and more dispersed fragments, whereas intact GM130-labeled Golgi complexes were observed in the cumulus cells (Fig. 6A, D, G). Staining for GM130 and P-GM130 did not overlap (Fig. 6C, F, I). At the GV stage, P-GM130 localized to a cortical domain (Fig. 6B, C). P-GM130 labeling persisted in the cortical domain in MI and MII stage oocytes, and increased in the ER as maturation progressed (Fig. 6E, H, J). No P-GM130 staining was detected in the polar body (Fig. 6H, I). These data indicate that during oocyte maturation, GM130 is stored in the ER in its phosphorylated form.

### CDC2 associates with SPDY at ERES

**Figure 7:**
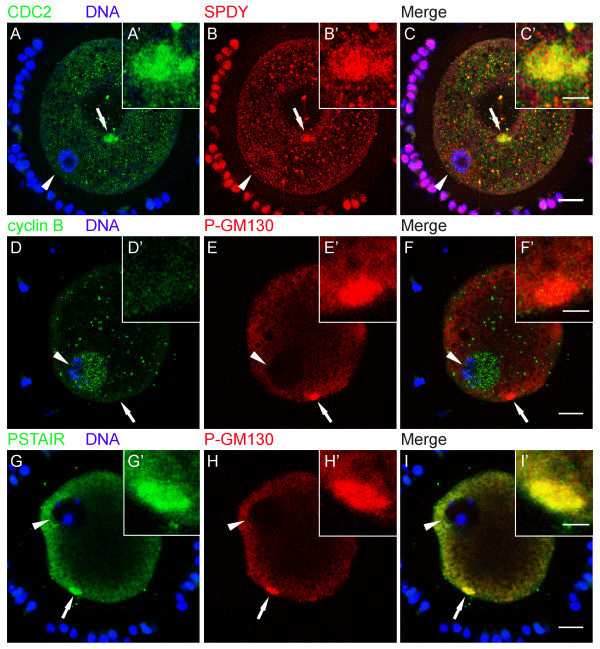
**CDC2 associates with SPDY at ERES**. (A-C) 0 h GV stage oocyte labeled for CDC2 (A, green), DNA (A, blue), and SPDY (B, red). Both CDC2 and SPDY localize to the same cortical domain (C). Note that the center of this oocyte is dented (the area that contains the arrow) causing the structure to appear in the middle of the oocyte, whereas it is located in the cortex. (D-F) 0 h GV stage oocyte stained for Cyclin B (D, green), DNA (D, blue), and P-GM130 (E, red). Cyclin B did not localize to the P-GM130-labeled cortical domain (F). (G-I) 0 h GV stage oocyte labeled for PSTAIR (G, green), DNA (G, blue), and P-GM130 (H, red). Spatial overlap of PSTAIR and phosphorylated GM130 staining (yellow) in a cortical domain is evident in the merged image (I). Images are Z-projections of 2 (A-C) or 3 (D-I) consecutive sections; scale bars represent 20 μm in C, F, and I, and 5 μm in C', F', and I'. Arrows indicate the region of the oocyte that is shown enlarged in the insets (A'-I'). Arrowheads denote the position of the GV.

The activity of CDC2 during cell division has been studied most extensively as MPF, a complex of CDC2 and cyclin B. Recently, evidence has been obtained that CDC2 activity in porcine oocytes can alternatively be controlled by SPDY, a protein with no homology to cyclin B [19]. Here, we used a combination of staining approaches to identify the regulatory component of CDC2 during early phases of maturation. Interestingly, we found that SPDY was present in CDC2-positive ERES in 0 h GV oocytes (Fig. 7A–C). Since simultaneous labeling of oocytes for cyclin B and CDC2 was not possible due to the same host species of cyclin B and CDC2 antibodies, we used an alternative approach in which we identified ERES by the presence of P-GM130. Oocytes (0 h, GV) double-labeled for P-GM130 and cyclin B showed no cyclin B in P-GM130-labeled ERES (0/21). Instead, about half of the oocytes (10/21) showed clear cyclin B labeling in the GV (Fig. 7D–F). Consistent with these observations, anti-PSTAIR antibodies, which do not detect CDC2 when bound to cyclin B [23], labeled P-GM130-containing ERES (11/18), whereas PSTAIR was not observed in the GV of any of the oocytes examined (0/18; Fig. 7G–I). These results indicate that CDC2 at ERES associates with SPDY, but not cyclin B, at the onset of oocyte maturation.

## Discussion

Here, we show that the cell-cycle regulating protein CDC2 transiently associates with a cluster of ERES at the onset of oocyte maturation, and propose that this association is involved in Golgi retention. Detailed microscopical analysis of large numbers of oocytes showed that during the initial phases of in vitro maturation, CDC2 localized to a large (~7 μm diameter) structure, that was exclusively observed in the cortex, in about a quarter of GV oocytes (Fig. 1A, F). The ERES marker SEC23 colocalized with CDC2 in about half of these cortical domains (Fig 2A–C), demonstrating that at least some of these CDC2-positive compartments consist of active ERES. A morphologically identical domain labeled for the CDC2 target protein P-GM130 was observed in ~80% of 0 h GV stage oocytes (Fig. 6J), and almost all CDC2-labeled cortical domains also labeled for P-GM130. In addition to this morphological likeness, only one P-GM130-labeled domain was observed per oocyte and this domain was always found in a cortical position. These identical features of SEC23, CDC2, and/or P-GM130-labeled domains indicate that all of these domains were composed of ERES, albeit mostly inactive ERES, since the majority was devoid of SEC23 labeling.

Membrane trafficking, and thus association of SEC23 with ERES, is inhibited during mitosis in somatic cells [12-14], which may in part be due to disassembly of ERES [18]. Upon disassembly, ERES-resident proteins disperse into the ER where they remain until the ERES are reformed at the start of interphase [18]. Our results support this view, since P-GM130 disperses from the ERES-containing domain into the ER prior to GVBD (Fig. 5C, E and fig. 6B, E, H). However, P-GM130 localization at the cortical domain was observed in >80% of oocytes at MI and MII (Fig. 6J). No difference in either morphology or position within the oocyte could be observed throughout maturation using P-GM130 as a marker for this domain (Fig. 6). Therefore, it can be inferred that the P-GM130-labeled domain is composed of ERES throughout maturation. When compared to ERES described in most somatic cells and bovine oocytes [6,18], the cortical domain in porcine oocytes is exceptionally large. Since SEC23 was not found outside the cortical domain (Fig. 2B), we conclude that most if not all ERES are clustered together in the cortex at this stage. The presence of this single cluster of ERES has not been reported before and appears to be a unique feature of oocytes.

During pre-GVBD IVM, the percentage of GV oocytes containing CDC2-labeled ERES decreased significantly (Fig. 1D). When the onset of maturation was prevented by isolating COCs in the presence of the adenylate cyclase-stimulating drug forskolin, the occurrence of CDC2-labeled ERES was significantly decreased (Fig. 5A), demonstrating that CDC2 translocation to and accumulation at ERES is initiated upon release from meiotic inhibition. This CDC2 translocation may therefore constitute the first sign of meiosis resumption. CDC2 accumulation at ERES was transient since the occurrence of CDC2-labeled ERES increased within 2 h after release from forskolin-maintained meiotic arrest, and subsequently declined to a much lower level after 18 h of maturation (Fig. 5B, D). CDC2-labeled ERES could not be detected in oocytes that had undergone GVBD, indicating that CDC2 accumulation, and hence CDC2 activity, at ERES is specific for pre-GVBD stages. Transient localization of CDC2 at ERES would also explain the discrepancy between the occurrence of the CDC2- (~25%; Fig. 1F) and the P-GM130-labeled (~80%; Fig. 5C and 6J) ERES cluster in immature oocytes. These results corroborate the proposition that stage dependent translocation of CDC2 underlies specific spatial and temporal control of CDC2 activity within oocytes [34-36].

It is evident from studies on somatic cells that CDC2 is involved in fragmentation and partitioning of the Golgi during early phases of mitosis [27]. Golgi fragmentation is initiated by CDC2-dependent phosphorylation of the membrane-resident Golgi protein GM130 [17], followed by vesiculation of the collapsing cisternae [37]. In our experiments, the presence of forskolin during oocyte isolation did not change the occurrence of P-GM130-labeled ERES at 0 h (Fig. 5C, E), indicating that P-GM130 accumulation at ERES precedes meiosis resumption. The percentage of GV oocytes with a P-GM130-containing ERES cluster declined from 80% in forskolin-inhibited 0 h matured oocytes to 35% at 18 h of maturation after forskolin removal (Fig. 5C). When oocytes were matured for longer intervals, i.e. 24 or 44 h, the percentage of oocytes showing a P-GM130-containing ERES cluster equaled that of the 0 h controls (Fig. 6J), whereas CDC2 did not localize at ERES clusters in MI (24 h) and MII (44 h) oocytes. These data indicate that the transient presence of CDC2 is closely followed by a transient absence of P-GM130 at ERES, prior to GVBD. Given the role of CDC2 in controlling ERES disassembly at the onset of mitosis [18], we propose that CDC2 controls dispersion of P-GM130 from ERES into the reticular ER.

Two models on Golgi partitioning during cell division are currently prevalent in literature. One model claims that Golgi inheritance occurs independently from the ER by association of Golgi-derived vesicles with the developing spindle, which facilitates subsequent equal partitioning of these Golgi fragments during telophase [7]. In the other model, known as the ER-dependent model, Golgi proteins redistribute into the ER during metaphase and Golgi stacks are reformed in daughter cells upon reinitiation of ER export [8]. In our experimental model, most oocytes contained a P-GM130-labeled ERES cluster immediately after isolation and at MI and MII (Fig. 6J), whereas P-GM130 was completely absent in the region surrounding the metaphase plates at both MI and MII (Fig. 6E, H). Staining of the non-phosphorylated form of GM130 in the oocyte showed an increasingly dispersed pattern as maturation progressed (Fig. 6A, D, G), and P-GM130 staining in the ER increased relative to staining in ERES (Fig. 6B, E, H), suggesting that phosphorylation of GM130 and redistribution of P-GM130 into the ER continues after meiosis resumption. Phosphorylation of Golgi-resident GM130 presumably triggers efficient translocation of this protein into the ER, because the distributions of GM130 and P-GM130 were mutually exclusive in porcine oocytes (Fig. 6C, F, I). Taken together, these results indicate that Golgi inheritance in oocytes occurs through redistribution of Golgi components into the ER, and thus support an ER-dependent model in oocytes.

Our data indicate that CDC2 at ERES was not complexed with cyclin B (Fig. 7D–I). Instead, we observed SPDY at CDC2-labeled ERES (Fig. 7A–C), suggesting that SPDY regulates CDC2 activity at ERES. Although we cannot conclude directly from our data that SPDY associates with CDC2 at ERES, additional evidence that SPDY may be a key regulatory protein of CDC2 upstream of MPF in porcine oocytes is provided by the observation that (1) CDC2/cyclin B activity can only be detected in a histone H1 kinase assay after 18–24 h of maturation [38-41] and that (2) injection of SPDY mRNA into oocytes accelerates oocyte maturation by stimulating MPF [21].

Our identification of a specialized cortical ERES-containing domain prompted us to compare it to similar structures described for other species. Morphologically, this cortical domain resembles Organized Smooth ER (OSER), which consists of stacked membrane arrays [42]. In our study, the ER-resident protein calnexin could not be detected in the CDC2-containing domain (Fig. 3G–I), indicating that this domain does not consist of reticular ER. In *Xenopus laevis *oocytes, cyclin B was found to localize to annulate lamellae, which may constitute one of the forms of OSER in immature oocytes [29]. Our studies show that neither the annulate lamellae marker NUP153 (Table 1, fig. 4J), nor cyclin B (Fig. 7A–C), localize to the cortical domain in immature porcine oocytes, suggesting that this domain does not consist of OSER or annulate lamellae. Primordial oocytes of several species contain an aggregate of organelles, the Balbiani body, which resembles the CDC2-labeled domain in size and in that there is only one of these bodies per oocyte. The Balbiani body contains components of several organelles including mitochondria, Golgi, and ER, and dissipates after the primordial stage [43]. P-GM130-labeled cortical domains were devoid of markers for intact Golgi (Fig. 6A, D, G) and mitochondrial markers (Table 1, fig. 4D–I), showing that the cortical domain and the Balbiani body are unrelated structures.

Based on observations in mitotic cells and our data from porcine oocytes, we hypothesize that prior to meiosis resumption, GM130 at the Golgi is phosphorylated by CDC2 [17,27] and subsequently transferred to ERES. Upon initiation of oocyte maturation, CDC2 activity at ERES may result in dissociation of SEC23-containing coats and consequent cessation of vesiculation [18,44]. Other protein complexes that control ERES function may also be phosphorylated [45], allowing dispersion of P-GM130 into the reticular ER, as observed for the ERES transmembrane marker Yip1A-GFP in CHO cells [18]. This mechanism could prevent recycling of GM130 back to the fragmenting Golgi and in this way facilitate complete Golgi breakdown. As pre-GVBD maturation proceeds, CDC2 is transported away from ERES (Fig. 1F), which may result in the renewed recruitment of P-GM130 to ERES (Fig. 6J). Since reformation of Golgi complexes at ERES has been observed in somatic cells [12-14], storage of P-GM130 in a cluster of ERES may allow for highly efficient and local reformation of the Golgi apparatus once the metaphase block of ER export is lifted after fertilization.

## Conclusion

In this study, we have observed a novel domain in the cortex of porcine oocytes that comprises a cluster of ERES. We found that the well-known meiotic regulator CDC2 transiently localizes to this domain during pre-GVBD maturation, immediately after the oocyte is released from the inhibitory influence of the follicular environment. Our data further suggest that CDC2, in conjunction with its regulatory protein SPDY, plays a role in regulating storage of structural Golgi elements at this ERES cluster. The early role of the CDC2/SPDY complex described here adds to the available evidence [21] that points to a role for CDC2/SPDY upstream of MPF during oocyte maturation. Finally, these findings demonstrate that pre-GVBD maturation comprises not only a set of changes in chromatin configuration [46], but also controlled and highly local events within the cytoplasm of the oocyte, that may be important in regulating the secretory system during the meiotic divisions.

## Methods

### Reagents and antibodies

All chemicals were purchased from Sigma Chemical Co. (St. Louis, MO, USA), unless otherwise indicated. The following antibodies and reagents were used (concentration or dilution and catalog number in brackets): mouse monoclonal anti-CDC2 (1 μg/ml; sc-54) and goat polyclonal anti-SEC23 (2 μg/ml; sc-12107) from Santa Cruz Biotechnologies (Santa Cruz, CA, USA); rabbit polyclonal anti-Calnexin (1:250; SPA-860) from Stressgen Biotechnologies (San Diego, CA, USA); mouse monoclonal anti-PSTAIR (1 μg/ml; ab10345, which is directed against EGVPSTAIREISLLKE, a conserved region in cyclin-dependent kinases (CDKs) [23,47]) and rabbit polyclonal anti-gamma-tubulin (1:1000; ab11321) from Abcam (Cambridge, UK); mouse monoclonal anti-NUP153 (4 μg/ml; MMS-102P) from Covance (Berkeley, CA, USA); mouse monoclonal anti-GM130 (1 μg/ml; G65120) and mouse monoclonal anti-cyclin B1 (5 μg/ml; 554179) from BD Biosciences (San Jose, CA, USA); rabbit polyclonal anti-SPDY (2.2 μg/ml; NB100–2521, directed against a conserved region that is present in both isoforms) from Novus Biologicals (Littleton, CO, USA); Mitotracker Deep Red (200 nM; M22426), goat anti-mouse IgG alexa488, goat anti-rabbit IgG alexa568, and rabbit anti-goat IgG alexa488 (20 μg/ml; A11029, A11036, and A11078, respectively) from Molecular Probes (Eugene, OR, USA); donkey anti-mouse IgG Cy3 (6.25 μg/ml; 715-165-151) from Jackson ImmunoResearch Laboratories Inc. (West Grove, PA, USA); rabbit polyclonal anti-P-GM130 (1:100; detects GM130 phosphorylated on serine 25 [27]) was a generous gift from Dr. Martin Lowe (University of Manchester, UK).

### Collection, culture, and assessment of porcine cumulus oocyte complexes

Cumulus-oocyte complexes (COCs) were collected from sow (*Sus scrofa*) ovaries, obtained from a slaughterhouse, by aspiration of 3–6 mm follicles [48] and subsequently selected using well established morphological criteria [49]. In vitro maturation (IVM) was performed as previously described [48], with the exception that media were not covered with oil. Briefly, COCs were collected in HEPES buffered M199 and washed in M199 (Gibco BRL) supplemented with 2.2 mg/ml NaHCO_3_, 0.1% (w/v) polyvinylpyrrolidone (PVP), 100 μM cysteamine, 75 μg/ml potassium penicillin G and 50 μg/ml streptomycin sulphate (oocyte maturation medium; OMM) [49-51], and equilibrated in a CO_2 _incubator (38.5°C; 5% CO_2_) for at least 2 h before use. Selected COCs were cultured at 38.5°C under 5% CO_2 _for 22 h in OMM supplemented with 0.05 IU/ml recombinant human FSH (rhFSH; a kind gift from Organon, Oss, the Netherlands). When COCs were cultured beyond 22 h, the medium was replaced with OMM without FSH (24 h and 44 h culture conditions). Based on overall morphology and DNA staining pattern that was assessed by confocal laser scanning microscopy (see below), in vitro matured COCs were subdivided into three categories: germinal vesicle (GV), meiosis I (MI; indicated by the presence of a metaphase plate, anaphase I and telophase I stages were included in this group), and meiosis II (MII; as indicated by the presence of a metaphase plate and the first polar body). Oocytes that could not be scored or showed an aberrant morphology and did not fit any of the above criteria were excluded from statistical analyses.

### Inhibition of meiosis resumption by forskolin treatment

COCs were kept in DMSO (control; 1:500) or 100 μM forskolin (50 mM forskolin in DMSO diluted 1:500) [31] during isolation and selection. Next, oocytes were denuded in DMSO or forskolin, fixed, and stained for CDC2 and DNA. In forskolin chase experiments, COCs treated with forskolin during isolation and selection were washed and cultured in OMM without forskolin for 0.5, 1, 2, or 18 h, and subsequently denuded and fixed. Finally, all groups were labeled for CDC2, P-GM130, and DNA, and the presence of structures was assessed using confocal laser scanning microscopy (as described below).

### Immunofluorescence staining for confocal laser scanning microscopy

After culture, COCs were washed in 80 mM PIPES, 5 mM EGTA, 2 mM MgCl_2_, pH 6.8, supplemented with 0.3% (w/v) PVP (PEM-PVP) at 37°C. To minimize mechanical stress that accompanies denudation procedures, but maintain sufficient antibody penetration after fixation, oocytes were partially denuded by gentle pipetting in PEM-PVP supplemented with either 0.01% (w/v) pronase (for < 22 h cultured oocytes), or 0.1% (w/v) hyaluronidase (for ≥ 22 h cultured oocytes). After washing in PEM-PVP at 37°C, oocytes were fixed in freshly prepared PEM-PVP containing 4% (v/v) paraformaldehyde (PF; Electron Microscopy Sciences, Hatfield, PA, USA) at room temperature (RT) for 1 h. Fixed oocytes were stored in PEM-PVP containing 1% (v/v) PF at 4°C for up to one week. For immunolabeling, oocytes were washed twice in PBS (0.1 M; pH 7.4) containing 0.3% (w/v) PVP (PBS-PVP) and once in PBS containing 0.1% (w/v) saponin (PBS-S) for 5 min. Aspecific binding sites were blocked using 1% (w/v) BSA and either 2% (v/v) normal goat serum, or 2% (v/v) normal horse serum (when goat polyclonal anti-SEC23 was used), both from Vector Lab (Burlingame, CA, USA), in PBS-S (blocking buffer) supplemented with 100 mM glycine for 2 h at RT or overnight at 4°C. Subsequent immunolabeling steps were performed sequentially in blocking buffer for 1 h at RT and followed by three rinses in PBS-S for 10 min each. Primary and secondary antibody dilutions were centrifuged at 100,000 g for 1 h before use. As negative controls, all experiments included 5–10 oocytes that were incubated with purified mouse IgG combined with rabbit IgG or normal goat serum matching the host species of primary antibodies used as appropriate. Control IgG concentrations and serum dilutions were identical to primary antibodies in the same experiment. DNA was labeled with 10 μM TO-PRO-3 iodide (Molecular Probes) in PBS-S for 20 min. After a final three washes in PBS-S, oocytes were mounted in a 0.12 mm 8 well Secure-Seal Spacer (Molecular Probes) on a coverslip, covered in Vectashield (Vector Lab), and sealed with a microscope slide (Superfrost Plus; Menzel, Braunschweig, Germany).

### Image acquisition and analysis

Images were obtained through a 40× oil immersion objective (N.A. 1.3) using a BioRad Radiance 2100 MP confocal system (Zeiss/BioRad, Hertfordshire, UK), equipped with 488, 543, and 637 nm lasers, or a Leica TCS SP2 confocal system (Leica Microsystems GmbH, Wetzlar, Germany), equipped with 488, 568, and 633 nm lasers. Dual and triple channel images were obtained by sequential scanning. ImageJ (NIH; ) image analysis software was used for qualitative analysis of images. Laser power and acquisition settings were adjusted to produce submaximal pixel values in the oocyte and settings used to image control IgG stainings were matched to the highest settings used to image primary antibody staining in the same experiment. Consecutive confocal sections were taken at 4 μm intervals. Scoring of the structures described in this paper was performed by sequential scanning through oocytes from top to bottom in live view mode and noting the presence or absence of a structure. When a structure was present, sections through both structure and DNA were taken, whereas only DNA-containing sections were taken in oocytes that did not contain a structure. Images were selected based on the presence of DNA and a structure within 6 consecutive sections to clearly show both maturation stage and presence of a structure in a single image. Background subtraction and contrast/brightness enhancement (up to ~20% enhancement using the maximum slider in imageJ) were performed on stacks of images, followed by maximum intensity Z-projection of consecutive sections to include both the structure and the region of the oocyte that contained chromatin. Contrast/brightness enhancement of IgG controls was identical to images from the same experiment.

### SDS-PAGE and Western blot

Oocytes were completely denuded by vortexing for 15 min in PBS-PVP containing protease inhibitor (Roche Molecular Biochemicals, Almere, the Netherlands). Denuded oocytes were separated from cumulus cells by two consecutive washes. Leftover cumulus cells were collected by centrifugation of the cumulus-containing suspension for 15 min at 16,000 g, and oocytes were collected by centrifugation for 5 min at 16,000 g. After removal of the supernatant, the cumulus cells and oocytes were snap-frozen in liquid nitrogen and stored at -20°C. Positive controls consisted of HeLa cells that were grown to near confluence and collected by centrifugation in PBS containing protease inhibitor (Roche). Cells were resuspended in 62.5 mM Tris-HCl, 2% SDS, and 10% glycerol (SDS sample buffer) to a concentration of 5 × 10^6 ^cells/ml and snap-frozen in liquid nitrogen. After addition of SDS sample buffer to oocyte and granulosa cell samples, all samples were incubated at 100°C for 5 min. Proteins were separated on 10% polyacrylamide gels (30 μl/slot) by SDS-PAGE and transferred to Polyvinylidene Fluoride membranes (Hybond-P; GE Healthcare Amersham Biosciences Europe Gmbh, Freiburg, Germany), which were subsequently blocked in PBS containing 0.1% Tween-20 (PBST) supplemented with 5% (w/v) non-fat milk powder (Protifar; Nutricia, Zoetermeer, the Netherlands), and incubated in antibody dilutions in PBST containing 0.5% (w/v) Protifar. Primary antibodies were probed using horseradish peroxidase-conjugated goat anti-mouse antibodies (Jackson ImmunoResearch Laboratories Inc., Westgrove, PA, USA) and detected by Supersignal west pico chemiluminescent substrate (Pierce Biotechnology, Rockford, IL, USA).

### Statistical analysis

Data are presented as weighted mean percentage ± weighted SEM of oocytes, unless otherwise indicated. Statistical analysis was performed on the original dichotomous data in SPSS 12.0 (SPSS Inc., Chicago, IL, USA) using chi-square tests unless otherwise indicated.

## Authors' contributions

JJH carried out the immunofluorescence and western blot studies, performed statistical data analysis, and drafted the manuscript. WS, BC, DRG, and BAJR participated in design of the study, and helped with data interpretation and critical evaluation of the manuscript. TH conceived of the study, participated in its design and coordination, and helped draft the manuscript. All authors read and approved the final manuscript.
